# The Role of the Gut Microbiome in Cow’s Milk Allergy: A Clinical Approach

**DOI:** 10.3390/nu14214537

**Published:** 2022-10-28

**Authors:** Dafni Moriki, Maria Pilar Francino, Despoina Koumpagioti, Barbara Boutopoulou, José Ángel Rufián-Henares, Kostas N. Priftis, Konstantinos Douros

**Affiliations:** 1 Allergology and Pulmonology Unit, 3rd Pediatric Department, National and Kapodistrian University of Athens, 12462 Athens, Greece; 2 Department of Genomics and Health, Fundación Para el Fomento de la Investigación Sanitaria y Biomédica de la Comunitat Valencia (FISABIO), 46020 Valencia, Spain; 3 CIBER en Epidemiología y Salud Pública, 28001 Madrid, Spain; 4 Department of Nursing, National and Kapodistrian University of Athens, 11527 Athens, Greece; 5 Departamento de Nutrición y Bromatología, Instituto de Nutrición y Tecnología de los Alimentos, Centro de Investigación Biomédica, Universidad de Granada, 18071 Granada, Spain; 6 Instituto de Investigación Biosanitaria ibs. GRANADA, Universidad de Granada, 18071 Granada, Spain

**Keywords:** food allergy, cow’s milk allergy, gut microbiome, dysbiosis, probiotics, prebiotics

## Abstract

Cow’s milk allergy (CMA) is the most prevalent food allergy (FA) in infancy and early childhood and can be present with various clinical phenotypes. The significant increase in FA rates recorded in recent decades has been associated with environmental and lifestyle changes that limit microbial exposure in early life and induce changes in gut microbiome composition. Gut microbiome is a diverse community of microbes that colonize the gastrointestinal tract (GIT) and perform beneficial functions for the host. This complex ecosystem interacts with the immune system and has a pivotal role in the development of oral tolerance to food antigens. Emerging evidence indicates that alterations of the gut microbiome (dysbiosis) in early life cause immune dysregulation and render the host susceptible to immune-mediated diseases later in life. Therefore, the colonization of the gut by “healthy” microbes that occurs in the first years of life determines the lifelong health of the host. Here, we present current data on the possible role of the gut microbiome in the development of CMA. Furthermore, we discuss how gut microbiome modification might be a potential strategy for CMA prevention and treatment.

## 1. Introduction

Food allergy (FA) is characterized as an adverse immune reaction to specific food antigens. Its incidence has been rising during the last two decades, and it now represents a considerable health problem affecting nearly 5% of adults and 8% of children worldwide [1]. Cow’s milk allergy (CMA) is the most common FA in infancy and early childhood, with an estimated prevalence in children under three years ranging from 0.5% to 3% in developed countries [2,3,4,5]. However, higher estimates have also been reported, mainly in studies evaluating the self-reported prevalence [6]. It usually occurs within the first year of life with a mean age of onset of 3.9 months [7] and has a favorable prognosis as the majority of children develop tolerance by the age of four [8,9]. However, in a subset of patients, CMA does not resolve until adolescence and adulthood, and patients experience severe allergic reactions to even traces of milk [10].

**Figure 1:**
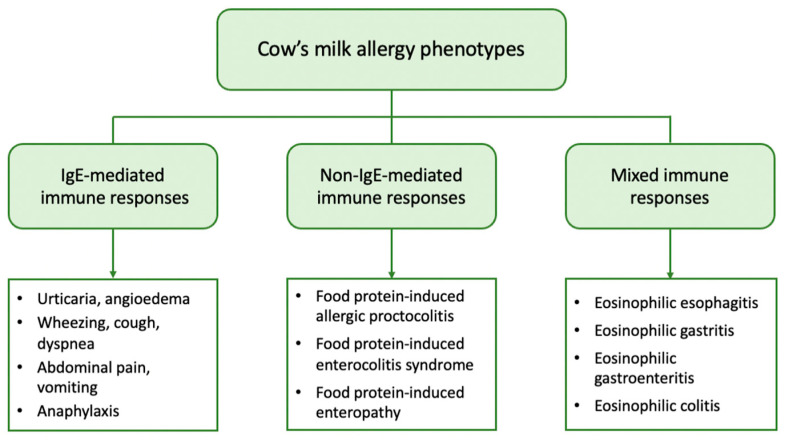
Clinical phenotypes of cow’s milk allergy.

Cow’s milk (CM) proteins are divided into two major classes, caseins (α1-, α2-, β-, and κ-casein) and whey proteins (α-lactalbumin and β-lactoglobulin), which account for 80% and 20% of total protein content, respectively. All proteins can be potential allergens and can induce both immunoglobulin E (IgE) and non-IgE-mediated immune responses, leading to a wide spectrum of clinical manifestations and distinct phenotypes (Figure 1). However, in the case of IgE-mediated CMA, multi-sensitization is more common [11].

Regardless of the underlying immune mechanism and clinical presentation, there is currently no effective treatment for CMA other than milk avoidance and emergency treatment in cases of accidental exposure. As a result, the quality of life of both the patient and caregivers is seriously affected due to the anxiety and fear of the unintentional ingestion of the offending CM protein, which can be potentially life-threatening. Oral immunotherapy (OIT) remains a choice for patients with IgE-mediated CMA who do not overcome the allergy [12]. However, its use is confined to a small number of patients and in centers with experience in the management of OIT and the capacity to deal with the often-severe adverse reactions. Furthermore, the administration of an extensively hydrolyzed casein formula (EHCF) or an amino acid-based formula (AAF) is required as a substitute for CM in most cases, leading to a significant increase in the cost of disease management [13]. Therefore, studies investigating the mechanism of achieving oral tolerance are of great importance.

Growing evidence suggests a central role of the human gut microbiome in the development of oral tolerance to dietary proteins. The human gut harbors an abundance of microorganisms and their corresponding genome, referred to as the gut microbiome. These complex communities have a symbiotic relationship with their host and are involved in key processes, including the development of the immune system. They interact with the innate and adaptive immune system and have tremendous potential to influence the physiology of the human body, both in health and disease [14,15,16]. Microbial colonization of the gut begins at birth and evolves in parallel with the host. However, there are several factors that can disrupt this process during the first years of life, leading to significant changes in the composition and function of the gut microbiome. Recent evidence suggests that early-life microbiome changes render the host vulnerable to immune-mediated disorders during childhood and later in life [17]. Understanding the process of early colonization of the gut and the complex way in which the microbiome interacts with the host immune system may therefore be the key to the prevention and treatment of immune-mediated diseases such as FA.

This paper aims to review the current state of knowledge regarding the potential role of the gut microbiome in the acquisition of oral tolerance and the pathogenesis of CMA. Furthermore, it aims to examine how modifying the microbiome composition could be a potential strategy for the prevention and treatment of CMA.

## 2. Clinical Phenotypes of CMA

From a clinical point of view, patients with CMA may present with a variety of symptoms linked to the immune mechanism underlying the allergic reaction. Based on the mechanism involved, CMA can be further classified into (a) IgE-mediated, which is caused by antibodies against milk proteins belonging to IgE, (b) non-IgE (or cell-mediated), where the cellular immune system, and especially T-cells, are responsible for the allergic reaction, and (c) mixed type when both IgE and immune cells are involved [18].

IgE-mediated CMA is the most common form and accounts for approximately 60% of all CM-induced allergic reactions [19]. It is caused by the production of specific IgE against CM proteins that bind to high-affinity IgE receptors (FcƐRI) on basophils and mast cells. Upon exposure, CM proteins are recognized by two or more specific IgEs that bind to FcƐRI with subsequent cross-linking of the receptor and activation of mast cells to release mediators such as histamine, tryptase, and platelet-activating factor [20]. These mediators cause vasodilation and elicit acute symptoms in the skin, gastrointestinal, respiratory, and cardiovascular systems. IgE-mediated reactions typically occur immediately after CM ingestion, or within 1 to 2 h, and may present as acute urticaria, angioedema, cough, wheezing, dyspnea, abdominal pain, vomiting, and hypotension. In severe cases, anaphylaxis, a systemic reaction that can be fatal, can also occur [18]. The diagnosis of IgE-mediated CMA is based on the combination of a compatible medical history and evidence of CM sensitization, i.e., the presence of specific IgE in skin mast cells (skin prick tests, SPTs) and/or in serum [21].

The non-IgE-mediated types of CMA have been far less studied. They encompass a wide range of disorders including food protein-induced allergic proctocolitis (FPIAP), food protein-induced enterocolitis syndrome (FPIES), and food protein-induced enteropathy (FPE). These disorders affect different segments of the gastrointestinal tract (GIT) and are distinguished by the delayed onset of symptoms, which can range from 2 h to several days after CM ingestion. They are caused by different immune cell-mediated mechanisms but have in common that they all lead to inflammation of the GIT. In these types of CMA, there is no evidence of CM sensitization, as no circulating specific IgE is detected [22,23]. However, a localized IgE response to the gut has been described [24].

FPIAP is one of the most common allergic diseases in infancy. It is characterized by the presence of mucus and blood in the stools of an otherwise healthy infant. Unlike other types of CMA, this clinical phenotype most commonly affects breastfed infants. It usually occurs in infants up to 3 months of age, and most of them develop tolerance within the first year of life. Diagnosis is based on clinical manifestations [23].

FPIES occurs less frequently. In two large prospective cohort studies from Israel and Spain, the cumulative incidence of CM-FPIES was 0.34% and 0.35%, respectively, in children during the first two to three years of life [25,26]. It mainly affects infants younger than nine months and is differentiated into acute and chronic FPIES. Infants with acute FPIES appear with multiple vomitings within a few hours of CM intake, which may be accompanied by pallor, lethargy, and diarrhea. In contrast, infants with chronic FPIES exhibit more chronic symptoms, such as vomiting, chronic diarrhea, and inadequate growth. Diagnosis in both cases is based on clinical criteria [27].

FPE is considered relatively uncommon, although its prevalence has not been thoroughly investigated. This disorder affects the small intestine and presents with malabsorption symptoms, such as chronic diarrhea, bloating, flatulence, anemia, and failure to thrive [28].

Mixed types of FA comprise a group of diseases generally referred to as eosinophilic gastrointestinal disorders (EGID). They are triggered by complex immune mechanisms with only partial involvement of IgE, leading to pathological eosinophilic infiltration of different parts of the GIT. Symptoms have a delayed onset and depend on the organs affected, as well as the extent of eosinophilic infiltration [29]. Eosinophilic esophagitis (EoE) is the most common disease, with CM and wheat being the main culprits. EoE in children presents with feeding difficulties, gagging, vomiting, and food refusal due to gastroesophageal dysfunction, and the diagnosis is based on endoscopic findings [22].

Atopic dermatitis (AD) is the most common chronic skin disease in childhood with an estimated prevalence of up to 20% [30]. It is a complex disease that is characterized by impaired skin barrier function due to various genetic, environmental, and immunologic factors. AD is usually the initial manifestation of the “atopic march” and predisposes to other allergic disorders such as FA, asthma, and allergic rhinitis [31]. Approximately one-third of children with moderate to severe AD develop FA, with CM being one of the main allergens [32]. However, the role of CM in the development of AD remains controversial, with many authors supporting the idea that CMA is a consequence of AD rather than AD being an expression or clinical phenotype of CMA [33]. In contrast, there are limited reports in the literature of isolated delayed-type eczematous reactions that typically occur within 6 to 48 h after food ingestion with eczematous flare-ups at AD-predisposed sites, suggesting a non-IgE-mediated pattern [32].

Lactose intolerance (LI) is a common gastrointestinal disorder that is caused by lactase deficiency and the inability to digest and absorb dietary lactose [34]. Lactose is the major carbohydrate in human and mammalian milk and is absorbed in the small intestine after first being hydrolyzed into D-glucose and D-galactose by the enzyme lactase [35]. Lactase expression is genetically programmed to decline gradually after weaning in about 70% of the world’s population [36]. As a result, lactase levels progressively decrease over time and its deficiency leads to lactose malabsorption and subsequent microbial fermentation of unabsorbed lactose in the colon [34]. Fermentation products, especially gasses, affect gastrointestinal function and may lead to the development of clinical symptoms, such as abdominal pain, flatulence, and diarrhea after consuming milk and dairy products. Despite similarities in clinical presentation with CMA, LI does not affect the immune system and is therefore not considered a FA. Moreover, it rarely manifests clinically in children younger than five years of age [37] and can be diagnosed by a variety of diagnostic methods, such as the hydrogen breath test [38].

## 3. Gut Microbiome and the Factors Influencing Its Development in Early Life

The GIT is colonized by a wide variety of microorganisms, mainly bacteria, which perform substantial metabolic, immunological, and gut protective functions. The structure of these complex microbial communities varies along the GIT and shows greater diversity in the colon where the bacterial density reaches 10^12^ colony-forming units (CFU)/mL [39]. Despite the large diversity, only four major phyla, Firmicutes, Bacteroidetes, Actinobacteria, and Proteobacteria, are represented in the human gut [40]. However, the exact taxonomic composition depends on many host-related factors, including genetic variation, age, diet, and geographic location, and thus varies significantly among healthy individuals [41].

The gut microbiome is acquired at birth and undergoes dynamic changes during the first years of life. In newborns it is initially dominated by Proteobacteria (e.g., *Escherichia*) and Actinobacteria (e.g., *Bifidobacterium*). Its composition is constantly changing and increasing in diversity throughout infancy and is largely mature by the age of two to three years. It then tends to acquire a more stable composition dominated by Firmicutes and Bacteroidetes and is characterized by distinct functions [42]. The ratio of Enterobacteriaceae to Bacteroidaceae, referred to as the E/B ratio, reflects maturation in the adult-type microbiome and decreases with age [43].

**Figure 2:**
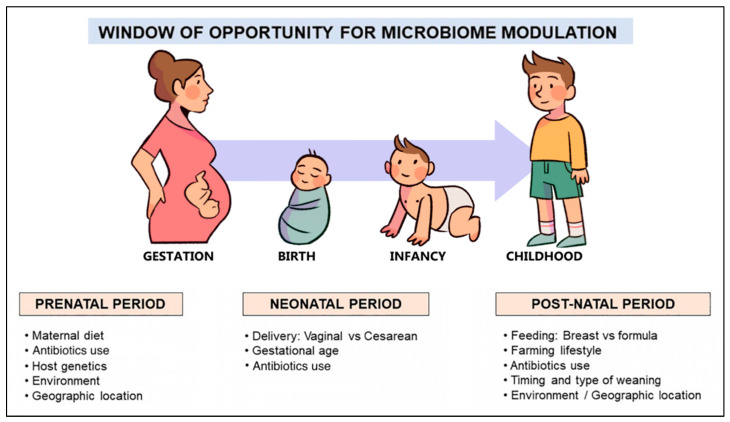
Factors shaping the human microbiome development in early life.

However, recent evidence suggests that the colonization of the gut may begin in utero, contrary to the widespread theory that the fetal environment is sterile [44,45,46]. Hence, the first 1000 days of life, or the time period from conception to two years of age, is considered the critical window for shaping the microbiome and regulating the immune system [47]. Mode of delivery, gestational age, breastfeeding, early exposure to antibiotics, and the timing and type of complementary feeding are the dominant factors (Figure 2) that influence the composition and function of the gut microbiome in this period and, therefore, determine the development of oral tolerance to different antigens [48,49,50,51].

The gut microbiome of infants born by cesarean section differs significantly from those born vaginally and thus exposed to their mother’s vaginal and fecal microbes. It is characterized by an enrichment of skin microbes such as *Staphylococcus* and *Streptococcus* [52], and a lower microbial diversity dominated by opportunistic pathogens such as *Enterococcus*, *Enterobacter*, and *Klebsiella* species, which are commonly prevalent in hospital settings [53]. The commensal bacteria that are typically detected in vaginally born infants (i.e., *Escherichia coli, Bifidobacterium*, and *Bacteroides*) are significantly lower, and colonization of the intestine by Bacteroidetes is delayed in cesarean-born infants [52,53]. These microbiome alterations have been associated with increased susceptibility to specific pathogens and atopic diseases [54,55,56].

Significant differences are also observed in the gut microbiome of infants according to gestational age. The microbiome of preterm infants is characterized by lower microbial diversity and a reduced abundance of strict anaerobes, particularly *Bifidobacterium*, compared to facultative anaerobes such as *Enterococcus*, *Streptococcus*, *Staphylococcus*, and various enterobacteria during the first three months of life. [57,58,59]. These alterations are related to several factors, including the immaturity of the immune response, the lack or delay of enteral feeding, and repeated and often prolonged exposure to broad-spectrum antibiotics, which disrupt the gut colonization process in early life and alter the integrity of the mucosal barrier [60]. As a result, microbial translocation and permeability to microbial components are increased, leading to a higher risk of life-threatening outcomes such as necrotizing enterocolitis and sepsis [60,61]. It is noted that gut microbiome diversity in preterm infants decreases with lower gestational age as well as with prolonged periods of antibiotic treatment and parenteral nutrition [59].

Breast milk is considered the optimal diet for infants and has a beneficial effect on the development of their gut microbiome. In addition to essential macro and micronutrients, it provides prebiotics that promote gut colonization with commensal bacteria and several bioactive factors that modulate host immune responses. As a result, breastfeeding is associated with lower incidence of allergic and autoimmune diseases [62]. Significant differences are observed in the gut microbiome of infants depending on the type of feeding. The microbiome of breastfed infants is dominated by species of *Bifidobacterium* and *Lactobacillus* which are known to have immunomodulatory properties and protect against allergies. In contrast, the microbiome of formula-fed infants contains a lower abundance of these beneficial bacteria and is dominated by the *Clostridium*, *Granulicatella*, *Citrobacter*, *Enterobacter*, and *Bilophila* species [42,63]. These differences persist in infants who are still breastfeeding at the age of 12 months and make their “microbiome age” appear younger than that of infants who no longer breastfeed. Therefore, sustained breastfeeding has a strong effect on the formation and succession of gut microbial communities during the first year of life and delays the development of an adult-like gut microbiome [42,63]. CMA, particularly allergic proctocolitis, may sometimes occur in breastfed infants. Although there will probably be differences in the gut microbiome between breastfed and formula-fed infants with CMA, there are currently no studies exploring this question.

Increasing evidence suggests that antibiotic administration disrupts the composition of the gut microbiome and impairs its function [64]. Early antibiotic exposure in infants is associated with reduced diversity and altered composition characterized by a depletion of *Bifidobacterium* and a marked increase in the Proteobacteria phylum [65,66]. Although weaker, similar alterations have also been found in the microbiome of infants whose mothers received antibiotics before delivery [53,65]. Of note, the effect of caesarean section on the infant’s gut microbiome is quite similar to that of maternal antibiotic use during pregnancy [53].

The Introduction of complementary foods into the diet of infants results in significant changes in their gut microbiome. Solid foods gradually lead to the establishment of an adult-type microbiome dominated by the phyla Bacteroidetes and Firmicutes, represented mainly by the genera *Bacteroides*, *Faecalibacterium*, *Clostridium*, and *Ruminococcus* [42,48]. In addition to compositional changes, the introduction of complementary foods is also accompanied by functional changes in the microbiome. In particular, it induces a sustained increase in the production of short-chain fatty acids (SCFAs) and promotes the enrichment of the microbiome in genes encoding polysaccharide breakdown, vitamin biosynthesis, and xenobiotic degradation [67].

In conclusion, studies have shown that the most “beneficial” gut microbiome in infancy is that of exclusively breastfed, vaginally delivered term infants which are characterized by the highest abundance of *Bifidobacterium* and the lowest amounts of *Clostridium difficile* and *Escherichia coli* [68].

## 4. The Role of the Gut Microbiome in the Development of FA

Recent data indicate that the composition and metabolic activities of the gut microbiome are inextricably linked to the development of oral tolerance, which is defined as an active process of local and systemic immune unresponsiveness to food antigens [69]. Studies in animal models revealed that germ-free mice were more likely to be sensitized to CM proteins than those whose gut was colonized with microbes, suggesting a protective effect exerted by the gut microbiome [70,71]. Remarkably, the transfer of fecal samples from healthy infants to germ-free mice reduced the risk of CM sensitization and the occurrence of anaphylaxis when exposed to beta-lactalbumin [72]. These findings suggest that the commensal microbial community drives the host towards the development of oral tolerance. However, the exact mechanism by which this is achieved remains to be further elucidated.

According to accepted theory, the process of oral tolerance occurs in the gut-associated lymphoid tissue (GALT) and involves the recognition of dietary antigens by dendritic cells and the induction of regulatory T cells (Tregs), as well as regulatory B cells (Bregs). Food antigens that cross the epithelial barrier are presented to naïve T cells of the mesenteric lymph nodes by specialized populations of dendritic cells in the presence of retinoic acid and TGF-β. This leads to the robust induction of antigen-specific FOXP3^+^ Tregs, which suppress the activation and differentiation of naïve T cells to Th types and have various anti-inflammatory roles. Suppression of Th2 responses also involves Bregs that contribute to oral tolerance by producing specific IgG4 [73].

Experimental research has shown that the interplay between the gut microbiome and the immune system is crucial for the production of Tregs which are necessary for the establishment and maintenance of oral tolerance. The colonization of the intestine of mice with specific bacterial strains, such as the *Bifidobacterium* and *Clostridium* species, was shown to have a protective effect on food allergen sensitization by induction of mucosal Tregs [74,75]. There are also studies denoting that commensal bacteria, particularly the Clostridia class, are likely to suppress FA through bacterial fermentation products, such as SCFAs, that promote the regulatory activity of dendritic cells and result in the induction of Tregs [76,77,78].

In addition to the induction of antigen specific Tregs, there is evidence that commensal microbes accelerate the development of oral tolerance through their protective effect on the intestinal epithelial barrier [79]. Indeed, some *Clostridium* species have been found to stimulate innate lymphoid cells to produce IL-22, which enhances epithelial barrier integrity and reduces intestinal permeability to protein antigens [80].

Another factor that has emerged as a crucial determinant for the protection and homeostatic regulation of the intestinal mucosal epithelium is the production of secretory IgA (sIgA), which is the major immunoglobulin on mucosal surfaces. Indeed, reduced fecal IgA levels and altered gut microbiome composition have been associated with food sensitization and an increased risk of anaphylaxis in mice [81]. The main function of sIgA, referred to as immune exclusion, is to limit the access of pathogens and food allergens to the mucosal barrier, and thus prevents their spread to the systemic compartment [82]. In addition, it regulates the balance of gut microbes by favoring the maintenance of beneficial members and the removal of opportunistic pathogens, which in turn prevents the absorption of pathogens and toxins [83,84]. There is evidence that mucosal secretion of sIgA is partially controlled by the gut microbiome and even that specific microbial species can induce its production to a different extent [84]. As a result, the composition of gut microbes strongly influences sIgA production and, consequently, the development of oral tolerance.

These data demonstrate a causal role for the gut microbiome in protecting against allergic responses to food antigens. Consequently, alterations in its composition, collectively referred to as dysbiosis, lead to immune dysfunction and predispose the host to the development of FA.

## 5. Association of Early Life Microbiome with Atopic Diseases

The prevalence of allergic diseases has increased significantly in recent decades, placing a substantial burden on health care systems in Western societies [85]. This increase has been attributed to environmental and lifestyle changes such as relocation from rural to urban settings, diets high in fat and protein, and the widespread and increasing use of antibiotics [86] that limit exposure to microbes in early life and result in the loss of gut microbial biodiversity [87,88]. In particular, antibiotic administration during early life has been associated with the development of FA, asthma, and allergic rhinitis later in life [89,90,91].

It is well established that the early microbial colonization of the gut strongly influences lifelong health through its close and decisive interaction with the immune system [92,93]. Reduced gut microbial diversity in early infancy has been correlated with subsequent food sensitization and increased risk of allergic diseases in childhood [94,95,96,97]. The underlying rationale is that frequent exposure to bacterial antigens may facilitate the establishment of immune regulation. However, when trying to explain the increasing trend in allergic disorders, the prevalence of specific microbial taxa is considered more important than microbial diversity. Indeed, perturbations in the gut microbial community during the neonatal period have been associated with the development of atopic diseases later in life in various experimental models [80,98,99,100,101]. Furthermore, alterations in commensal microbial communities in early life are also correlated with the pathogenesis of allergic diseases in humans. For example, an increased E/B ratio, which is indicative of an immature microbiome, was observed early in life in infants who were later sensitized to food antigens [94].

In a large prospective cohort study of 319 infants, a selective reduction in the relative abundance of bacteria from the genera *Lachnospira*, *Veillonela*, *Faecalibacterium*, and *Rothia* was observed in samples obtained at 3 months of age from infants who developed wheezing and positive allergen SPTs at 12 months compared to those who did not. This selective reduction in bacterial taxa was also accompanied by reduced fecal acetate levels. However, these alterations were no longer detected in fecal samples obtained at 12 months of age, suggesting that the first 100 days of life represent a window of opportunity for immune regulation [102]. Therefore, understanding the gut colonization process and microbiome function in early life is likely to contribute to the further understanding of allergic diseases, particularly FA, as well as to the design of microbe-based therapies to prevent them.

## 6. Gut Microbiome in Children with CMA

Several studies have revealed that children with CMA present with an altered gut microbiome at the time of diagnosis. However, it is uncertain whether dysbiosis is the cause or consequence of disease, mainly because the precise molecular pathways through which altered gut microbiome can cause various diseases remain unknown [103].

Gut dysbiosis is characterized by an imbalance of gut microbial communities that leads to functional and metabolic changes and disrupts gut homeostasis [104]. There are three types of dysbiosis that usually coexist: (1) the loss of overall microbial diversity, (2) the loss of beneficial bacteria, and (3) the expansion of opportunistic pathogens [105]. Gut dysbiosis is considered to promote pro-inflammatory effects and immune dysregulation and is associated with several disorders, including FA [106].

Microbial diversity is widely recognized as a determinant factor in regulating the stability of gut ecology. A diverse gut microbiome can generally perform better and is more resilient to environmental changes [107]. However, in the case of CMA, there is a disagreement among authors as to whether increased microbial diversity has a protective effect [108]. This is because studies evaluating the diversity of the gut microbiome in infants with CMA at the time of diagnosis have produced conflicting results [109,110,111,112], and only one of them shows reduced diversity in allergic infants [112]. These findings suggest that the involvement of the gut microbiome in the development of CMA cannot be explained by a single factor, such as microbial diversity, but, rather, involves more complex interactions between different bacterial taxa and their metabolic consequences [108].

*Bifidobacteria* and *Lactobacilli* are common beneficial bacteria that are typically abundant in the gut of infants, especially those who are breastfed. They have been shown to have immunomodulatory effects and protect against atopy and allergic disease in murine models [74,113,114,115,116,117,118]. Thus, it has been hypothesized that the loss of these beneficial bacterial species from the infant’s gut causes dysbiosis and may have negative effects on gut microbiome function. Indeed, lower concentrations of *Bifidobacterium* have been recorded in the gut of infants with CMA, underscoring their critical role in the development of CMA [109,111]. The introduction of such microbes has long been proposed as a means of preventing sensitization and aiding immunotherapy [119,120,121,122]. The most common microbe that has been used for this purpose is *Lactobacillus rhamnosus* GG, which has been shown to promote intestinal and respiratory immunity [123,124] and accelerate oral tolerance in infants with CMA [120].

Undoubtedly, the Clostridia class from the Firmicutes phylum and its metabolites play a critical role in the development of FA. However, there are currently two opposing views on whether clostridia are beneficial or harmful [125]. On the one hand, there are experimental studies showing that certain *Clostridia* species can mediate immune responses through their fermentation products and thus promote homeostasis and health in the colon. These bacterial species produce SCFAs, especially butyrate and propionate, which are known to induce mucosal Tregs and accelerate oral tolerance to specific antigens [75,77,78]. Moreover, butyrate has been found to enhance the epithelial barrier and reduce the access of food allergens to the bloodstream [80]. In a prospective birth cohort study of 1133 children, higher levels of fecal SCFAs (especially butyrate and propionate) in early life were associated with reduced risk of food sensitization and less incidence of allergic diseases at school age [126].

On the other hand, it has been shown that butyrate produced by bacteria in the Firmicutes phylum can cause mucosal injury in newborn rabbits and thus possibly increase the permeability of the intestinal epithelium, resulting in higher concentrations of food antigens reaching the bloodstream [127]. In addition, an increased abundance of *Clostridium coccoides*, along with higher levels of butyrate, has been found in the gut of infants with CMA [110]. Another study also showed increased levels of *Clostridium coccoides* in children with non-IgE CMA who did not develop tolerance, leading to the conclusion that this bacterial species may be harmful and negatively affect the outcome of CMA [121]. It is likely that potential harmful effects are limited only to certain species and even strains of clostridia and their metabolites.

### 6.1. IgE-Mediated CMA

Studies evaluating the gut microbiome in infants with IgE-mediated CMA are rare and have yielded conflicting results. This is due to methodological issues and mainly to the use of different techniques for studying the microbiome. For example, culture-based techniques used in the past can only detect 30–50% of the bacteria that inhabit the gut, while the introduction of next-generation sequencing and genomic analysis allows us to identify more microbial species [41]. Furthermore, temporal variability in the human gut microbiome is a serious limitation in studies with a single measurement design [128].

In a prospective case-control study conducted in Spain, the gut microbiome of infants with IgE-mediated CMA was compared with that of healthy infants both at the time of diagnosis and after six months of EHCF consumption using conventional aerobic and anaerobic cultured-based techniques. At both time points, allergic infants appeared to have higher total bacterial counts, particularly of anaerobes, compared to healthy infants. Furthermore, the gut microbiome of allergic infants consisted of higher proportions of lactobacilli and lower proportions of enterobacteria and bifidobacteria after six months, but no significant differences were observed at baseline [109]. The same study team also examined the relative abundance of gut microbiome constituents at the baseline using fluorescent in situ hybridization combined with flow cytometry. They showed that *Clostridium coccoides* and *Atopobium* cluster species were significantly more common in the gut microbiome of allergic infants, but there were no differences in *Bifidobacterium*, *Lactobacillus*, and *Bacteroides*. In addition, allergic infants had higher fecal concentrations of SCFAs, such as butyrate, compared to healthy infants [110].

In a more recent cross-sectional study using next-generation sequencing, the gut microbiome of allergic infants was found to be significantly more diverse at the time of diagnosis compared to healthy infants. The taxonomic analyses revealed a reduction of Bifidobacteriaceae, Streptococcaceae, Enterobacteriaceae, and Enterococcaceae, and enrichment of Ruminococcaceae and Lachnospiraceae in allergic infants. Moreover, the gut microbiome of allergic infants consisted mainly of Bacteroidetes and Firmicutes taxa, which are known to predominate in the adult gut [111].

Finally, another prospective case-control study compared the gut microbiome of infants with CMA (both IgE and non-IgE mediated) at the time of diagnosis and after six months of treatment with a hypoallergenic formula using 16S rRNA sequencing. The authors found that infants with CMA had lower gut microbiome diversity and increased E/B ratio at the time of diagnosis compared to healthy controls. Of note, after six months of follow-up, there was no significant difference in the E/B ratio between the two groups, although the allergic infants had higher concentrations of Lactobacillaceae and lower concentrations of Bifidobacteriaceae and Ruminococcaceae than the healthy infants [112].

### 6.2. Non-IgE-Mediated

Results are less clear in the case of non-IgE-mediated types of CMA, not only because of the use of different microbiome analysis techniques, but also because of the wide heterogeneity of non-IgE-mediated types and the difficulties in establishing a correct diagnosis [122,129,130,131]. Nevertheless, there is evidence that food-allergic infants display specific microbial signatures related to the underlying immune mechanism (IgE-mediated and non-IgE-mediated), which could be used as potential biomarkers for the diagnosis of FA [131].

Of note, some preliminary results that have not yet been corroborated showed that, at the phylum level, children with non-IgE-mediated FA show a higher relative abundance of Proteobacteria in the gut compared to healthy controls [132]. Of the four phyla that predominate in the human gut, the Proteobacteria phylum is the most unstable over time and is considered the first line of response to environmental factors [133]. These bacteria are innocuous when present in small amounts, but under certain conditions they can induce inflammatory responses [134]. Hence, chronic enrichment of Proteobacteria in the gut indicates an imbalanced unstable microbial community structure and has been associated with several diseases [135,136].

## 7. CMA Treatment: Probiotics, Prebiotics, and New Therapeutic Strategies

Some children with CMA can tolerate extensively heated milk as well as small amounts of milk and dairy products. However, complete avoidance of CM proteins is currently the only treatment for patients with severe CMA. In these cases, children must avoid a variety of foods, which is challenging and stressful for both children and their caregivers, as even traces of milk can induce severe allergic reactions. In addition, milk and dairy products are essential components of children’s diets and prolonged abstinence may have harmful effects on their growth and nutritional balance [137,138]. As a result, a hypoallergenic formula (EHCF or AAF) must be administered as a substitute for CM, with a consequent increase in the cost of disease management. Hence, there is an apparent need to move from CM avoidance to active treatments [139].

Increasing evidence suggests that gut microbiome composition and function in early life are associated with CMA outcome. In a prospective study of 234 children with IgE-mediated CMA, enrichment of the gut microbiome with taxa from the Clostridia class and Firmicutes phylum at three to six months of age was correlated with CMA resolution at eight years of age [140]. These findings highlight the critical role of commensal bacteria in modulating host immune responses to dietary antigens and accelerating the acquisition of oral tolerance. Therefore, modifying the microbiome during early life could be a potential therapeutic strategy for CMA. Indeed, extensive research has been conducted on the effect of probiotics, alone or in combination with prebiotics, on the gut microbiome, with some studies yielding encouraging findings.

Probiotics are live microorganisms, mainly bacteria, that have significant health benefits for the host. The most common belong to two genera, *Lactobacillus* and *Bifidobacterium*, and when administered in appropriate doses, they maintain a balance in the microbiome composition and suppress the growth of pathogenic bacteria in the gut. They also strengthen the intestinal epithelial barrier by reducing intestinal permeability, inducing sIgA, increasing mucus thickness, and producing defensin [141]. Moreover, probiotics interact with immune system cells in the GIT and modulate immune responses, thus helping to prevent allergic diseases [142].

In a randomized control trial, the administration of an EHCF supplemented with the probiotic *Lactobacillus rhamnosus* GG resulted in a significantly greater number of children who reached oral tolerance and CMA resolution over a 36-month period compared to those who received the non-supplemented hypoallergenic formula [120]. In a previous study, this probiotic was found to increase the relative abundance of certain Clostridia species, including *Blautia*, *Roseburia*, and *Coprococcus*, which expanded butyrate production when administered in combination with EHCF in infants with IgE-mediated CMA [111]. On the contrary, a different randomized control trial failed to demonstrate that the use of an EHCF, supplemented with a probiotic blend (*Lactobacillus casei* CRL431 and *Bifidobacterium lactis* Bb-12), induces oral tolerance in infants with CMA [119].

Prebiotics are substrates selectively utilized by indigenous microbes resulting in beneficial changes in the composition and/or activity of the gut microbiome. They are abundant in human milk and exert their effect both directly through their interaction with the intestinal epithelium and indirectly through the metabolic products generated by bacteria upon their fermentation [143].

Administration of synbiotics (i.e., a combination of probiotics and prebiotics) also appears to be effective in non-IgE-mediated CMA. The use of an AAF supplemented with the combination of a prebiotic fructo-oligosaccharide and the probiotic strain *Bifidobacterium breve* M-16V in infants with non-IgE-mediated CMA improved the composition of the gut microbiome and its metabolic activities. In particular, a relative increase in several *Bifidobacterium* species was observed, combined with a decrease in numerous species of the *Lachnospiraceae* family and a decrease in the levels of branched chain SCFAs [121,122,144].

OIT is a potential therapy for patients with IgE-mediated CMA. The rationale behind this treatment is to induce desensitization through daily consumption of increasing amounts of CM. However, its efficacy is controversial, and it does not produce long-term tolerance in all patients. In addition, its use is limited to selected patients due to frequent side effects [12]. A recent randomized trial showed that co-administration of the probiotic *Lactobacillus rhamnosus* GG with peanut OIT enhanced treatment efficacy and induced immunological changes and oral tolerance in children with a peanut allergy [118].

Fecal microbial transplantation (FMT), which is the transfer of normal gut microbes from healthy individuals to patients in order to reconstruct their gut microbiome ecology, may be a potential therapeutic strategy for CMA in the future. FMT is already applied to the treatment of several diseases, including inflammatory bowel diseases and *Clostridium difficile* infections, and shows satisfactory results [145]. Moreover, studies in experimental models have revealed that transplantation of gut microbiome from healthy infants protects mice from CMA [72,80]. A recent study also showed that maternal FMT restored the gut microbiome of neonates born by caesarean section to a normal state similar to that of vaginally delivered infants, indicating that FMT can regulate the microbiome of infants and may be a potential therapeutic option [146]. However, the curative effects of FMT in the treatment of CMA require further investigation.

Despite the promising results of these treatments, none is yet fully effective and all present significant limitations and drawbacks. In the future, the modification of the gut microbiome through personalized nutrition will probably be the key to the prevention and treatment of CMA. To achieve this, however, we must first understand the microbial taxa involved in CMA and how these microbes interact with different foods (diet-microbiome interactions) [147].

## 8. Conclusions

CMA is a common allergic disease in infancy and early childhood that has a significant impact on the quality of life of children and their families. Growing evidence suggests a central role of the gut microbiome in the development of CMA through its close and decisive interaction with the immune system. Several studies have been conducted on the effect of various factors on the gut microbiome and how its modification through different methods in early life contributes to the prevention and treatment of CMA. However, there are currently no recommendations for clinicians, as we still have much to learn about the complex crosstalk between the gut microbiome and the immune system (which will shed light on the development of new strategies).

## Data Availability

Not applicable.
